# Modified Dachengqi Decoction Combined with Conventional Treatment for Treating Acute Exacerbation of Chronic Obstructive Pulmonary Disease: A Systematic Review Based on Randomized Controlled Trials

**DOI:** 10.1155/2013/323715

**Published:** 2013-04-03

**Authors:** Ruohan Wu, Zheng Fengjie, Yuhang Li, Sun Yan, Liu Miao, Wang Tan, Zhang Jinchao

**Affiliations:** School of Preclinical Medicine, Beijing University of Chinese Medicine, 11 North Sanhuan East Road, Chaoyang District, Beijing 100029, China

## Abstract

*Objective*. This study intended to systematically evaluate the effectiveness and safety of modified Dachengqi Decoction (MDD) combined with conventional treatment for treating acute exacerbation of chronic obstructive pulmonary disease (AECOPD). *Method*. An extensive search was performed within 6 English and Chinese electronic databases from inception to April 2012. Methodological quality was assessed according to Cochrane risk of bias assessment. Data were analyzed using Review Manager 5.1. *Results*. A total of 16 studies (involving 1112 patients) were included. The result showed that MDD and its modification combined with routine treatment were more effective in improving FEV_1_%pred, enhancing the significant effectiveness, reducing PCO_2_, and shortening duration of mechanical ventilation. Adverse events were reported in two trials with symptom of diarrhea, while no serious adverse effect was reported. *Conclusion*. Modified Dachengqi Decoction appears to be effective for treating AECOPD. However, more regular designed RCTs are needed because of insufficient methodological problems.

## 1. Introduction

Global strategy for the diagnosis, management, and prevention of chronic obstructive pulmonary disease (COPD) [1] for the first time revised the goal of the treatment of COPD into relief symptoms rapidly, and reduces risk of patients' health, such as recurrent episodes or rapid decrease of pulmonary function [2]. Therefore, how to control the symptoms and reduce the frequency of disease onset became the research emphasis in academic field.

COPD belongs to the category of “lung distention” in Chinese medicine. Syndrome of phlegm-heat obstructing in the lung is one of the most common syndromes in the acute stage. The main clinical features include yellow sputum, dyspnea, thirsty, and constipation [3]. “Interior and Exterior Relationship between the Lung and Large Intestine” is one of the most typical viscera correlation theories. It describes the corelationship of viscera, meridian, physiological relation, and pathological changes, which is the foundation for further clinical treatment of AECOPD by using purgative drugs. Many domestic and foreign researches [4, 5] found that COPD patients have digestive tract symptom such as abdominal distension, constipation besides cough, wheezing, phlegm, and dyspnea. So, it is viable to do research on using purgative decoction in the treatment of AECOPD. 

Dachengqi decoction, a representative recipe of dredging intestines in *Treatise on Febrile Diseases *(*Shang Han Lun*), has been widely used to treat Yangming Fushi Syndrome. It is composed by Rheum, *Magnolia officinalis*, immature bitter orange, and Mirabilite. In a recent study, it effectively treated critical patients with gastroenteric function disorder, and reduce the incidence and fatality of MODS [6]. 

This study aimed to determine the effects and safety of purgative decoction on pulmonary function, artery blood gas analysis, ventilator weaning time in patients with AECOPD by systematically evaluating the effectiveness of oral decoctions, or Chinese patent medicine based on Dachengqi Decoction plus conventional treatment compared with western medicine alone in the treatment of AECOPD. 

## 2. Materials and Methods

### 2.1. Search Strategy

We searched the Chinese literature from CNKI, CBM, VIP, WANFANG, and foreign literature from PubMed and Cochrane library. The searching was from the inception of the databases to April 2012. We utilized the medical subject headings “COPD” or “chronic obstructive pulmonary disease” and “Chinese medicine” in PubMed, Cochrane, while we use “COPD” or “chronic obstructive pulmonary disease” and “Dachengqi Decoction” or “Radix et Rhizoma Rhei” or “Natrii Sulfas” or “Fructus Aurantii Immaturus” or “Cortex *Magnoliae officinalis*” in Chinese database.

### 2.2. Inclusion Criteria

Inclusion criteria were the following: (1) RCTs in English or Chinese involving decoctions based on Dachengqi Decotion compared with placebo, no treatment, or conventional treatment without Chinese medicine as controls. (2) Patients must be aged 18 years or over and of any gender or ethnic origin. Patients are diagnosed with COPD in the severe stage with or without respiratory failure. COPD is defined as “The Global Initiative for Chronic Obstructive Lung Disease (GOLD)” which is pulmonary function includes FEV_1_%pred <80% and FEV_1_/FVC% <70% after using bronchodilator. (3) Primary outcome measures were pulmonary function (FEV_1_%pred, FEV_1_/FVC%), safety, and significant effectiveness based on clinical symptoms relief. The significant effectiveness was defined as the symptoms scores improvement rate ≥70% according to “the guide for clinical trials of new drugs” [7]. Clinical symptoms involved cough, cough-up phlegm, dyspnea, constipation, and wheeze. Secondary outcome measures were artery blood gas analysis (PO_2_, PCO_2_) and duration of mechanical ventilation. 

### 2.3. Data Extraction

Study characteristics included trial design, sample size, mean and standard deviation of participants' age, and history of COPD; severity of COPD differentiation of syndrome, methodological quality, intervention, outcome measures, treatment duration and follow-up period, and adverse events were extracted to a predefined form and checked by a second reviewer.

### 2.4. Risk of Bias

The methodological quality of the included studies was independently assessed by 2 authors using the Cochrane risk of bias assessment [8]. Assessment of Cochrane risk of bias consists of seven domains: (1) random sequence generation (2) allocation concealment (3) blinding of participants and personnel (4) blinding of outcome assessment (5) incomplete outcome data (6) selective reporting and (7) other bias. For each domain, evaluation was by denoting “yes”-adequate (low risk of bias); “no”: inadequate (high risk of bias); or “unclear”: unclear or not used (uncertain risk of bias) according to the descriptions of the method in each study. Any disagreement was resolved by discussion with a third reviewer.

### 2.5. Data Analysis

Meta-analysis was performed using RevMan 5.1. For categorical data, we used risk ratios (RR), while for continuous data, mean differences (MD) were calculated and expressed in effect value and 95% confidence (CI). Heterogeneity was calculated by *X* ^2^ and *I* ^2^ statistics. When heterogeneity inspection result showed significant heterogeneity (*P* < 0.05), we used random effects model, otherwise we applied fixed effects model.

## 3. Results 

### 3.1. Overview of Included Studies

**Figure 1:**
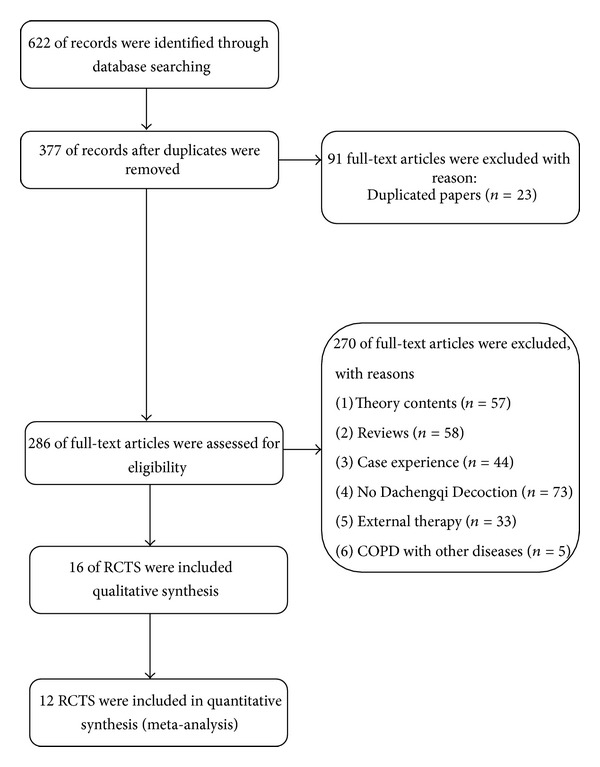
Study selection process.

We initially identified 622 citations, after screening for potential relevance, 16 full papers [9–24] were assessed for possible inclusion (Figure 1). All studies were conducted in China. The characteristics of the included studies are summarized in Table 1. The different compositions of Chinese herbal formula MDD are presented in Table 2. The 16 studies involved a total of 1112 acute COPD patients, and 12 studies were included in meta-analysis. All studies reported diagnosis standard. All 12 studies were about Chinese medicine combined with western medicine routine treatment compared with conventional treatment alone (Table 1). Fifteen studies [9–13, 15–18, 20–24] were about oral decoction combined with western medicine routine treatment. Two [14, 19] studies used Chinese patent medicine based on MDD. 

### 3.2. Assessment of Risk of Bias

Information of sequence generation was adequate for five studies at low risk of bias [9, 13, 18, 22, 23] and inadequate for seventeen studies with unclear risk of bias [10–12, 14–17, 19–21, 24]. All the five studies reported that they used random number table for sequence generation. Allocation concealment was not reported in all studies (no). Blinding of participants, physicians, and study personnel was not reported in these studies, but all studies were carried without placebo, so they were of high risk of bias. None of the studies reported lost to followup, withdraw and dropoff; thus, the risk of bias of incomplete outcome data over all studies was graded as unclear. As far as selective reporting was concerned, for we could not find any pre-defined outcome measurements in all the icluded studies we classified them as unclear risk of bias. Due to the limited number of included studies, we were not able to implement funnel plot. 

### 3.3. Outcome Measure

#### 3.3.1. Pulmonary Function

**Figure 2:**
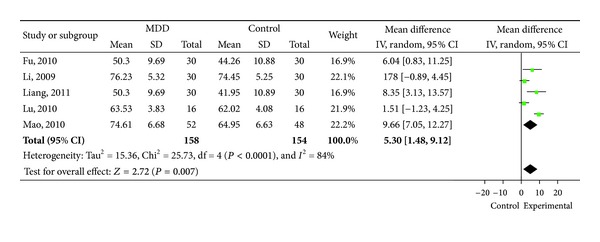
Forest plot of comparison: FEV_1_%pred (%).

**Figure 3:**
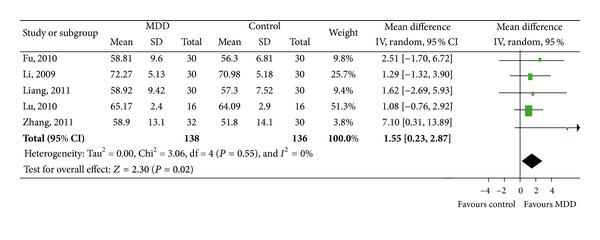
Forest plot of comparison: FEV_1_/FVC% (%).

FEV_1_%pred was reported in 5 articles [10, 13, 16, 18, 19] (Figure 2 ) and FEV_1_/FVC% was reported in 5 studies, respectively [10, 13, 16, 18, 24] (Figure 3). Significant differences showed in FEV_1_%pred. Decoction group concludes five trials (MD 5.3, 95%CI 1.48 to 9.12). Similar changes were shown in FEV_1_/FVC% (MD 1.55, 95%CI 0.23 to 2.87). 

#### 3.3.2. Significant Effectiveness

**Figure 4:**
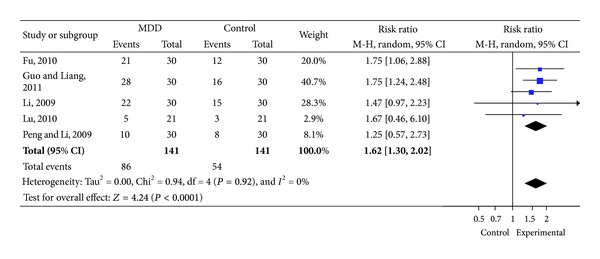
Forest plot of comparison: therapeutic effect of syndrome.

MDD group showed higher percentage of effectiveness when compared with non-MDD formula group (RR 1.62, 95%CI 1.3 to 2.02) [10, 11, 13, 18, 22] (Figure 4).

#### 3.3.3. Blood Gas Analysis

PO_2_ was reported in 6 studies [9, 11, 13, 18, 20, 21] (Table 3) and PCO_2_ was predicted in 5 studies [9, 11, 18, 20, 21] (Table 4). There was an improvement in PO_2_ and a reduction in PCO_2_ when comparing modified Dachengqi Decoction plus conventional treatment to conventional treatment alone. 

#### 3.3.4. Duration of Mechanical Ventilation

Duration of mechanical ventilation (days) was reported in 2 studies [9, 23]. Recovery time of MDD group was shorter than that of the conventional treatment group (MD −3.16d, 95%CI −3.9d to −2.43d). 

#### 3.3.5. Safety

Two studies [15, 19] reported adverse events. Both trials reported adverse reaction as diarrhea. Other trials did not report it.

## 4. Discussion

This study focuses on evaluating the effectiveness and safety of modified Dachengqi Decoction for AECOPD based on pulmonary function, blood gas analysis, and effective rates when compared with conventional treatment group. Based on the study of the sixteen studies, DMM may have positive effect on improving patients pulmonary function, improving the symptoms, enhancing the partial pressure of oxygen, decreasing the partial pressure of carbon dioxide, and shortening the duration of mechanical ventilation. As with any meta-analysis, heterogeneity must be considered. We found significant heterogeneity in the outcome measure for FEV_1_%pred, but the heterogeneity in the outcome measure for FEV_1_/FVC and clinical symptom relief was very low. 

Only two studies [15, 19] reported a total of three adverse events of diarrhea,which suggests that the MDD for COPD is well tolerated. However due to the incomplete evaluation, safety of DMM should be accepted more cautiously. The results need to be monitored rigorously in the future. 

Some systematic review [25, 26] has indicated a benefit of using Chinese herb such as oral ginseng formulae for the management of stable COPD which belongs to deficiency syndrome according to TCM (traditional Chinese medicine) theory. Also, there were many studies showing that Chinese medicine had become more and more important in treating COPD/AECOPD [27]. There have been many randomized controlled trials indicating that herbs can release clinical symptoms and improve quality of life. 

MDD is not available for it is not widely used in treating COPD/AECOPD, but some studies [5] found that COPD patients have digestive tract symptom such as abdominal distension, constipation besides cough, phlegm, and dyspnea. Thus, it is practical to use MDD in treating COPD which belongs to excess syndrome especially with constipation symptoms in TCM. The theory of “Interior and Exterior Relationship between the Lung and Large Intestine” is one of the most important theories in traditional Chinese Medicine which is of great value in the clinical practice. Also some studies found that “catharsis large intestine” can decrease T cells and enhance the number of serum T cells and affect the balance of CD4^+^ and CD8^+^ and can have effects on airway remodeling of lung in rats with COPD [28].

Also, there are several methodological limitations. First, all trials involved were of low quality. No study applied placebo as control; thus, the patients and physicians were not blinded. Although all the trials reported randomization, only 5 studies reported sequence generation and no study addressed the issue of allocation concealment. The quality of studies published in Chinese is majorly poor, and some scholars indicated that China generated virtually no negative studies at all [29]. Therefore, the findings of the meta-analyses should be interpreted with caution. Due to the time limitation, we did not contact the original authors, and further information for better evaluation of risk of bias was inadequate.

Second, all the included studies were published in Chinese journals, and all the results were positive. What is more, study number was not enough to implement funnel plot, so there might be a potential publication bias. We could not rule out the systematic error because the sample size of all studies was limited. So the clinical effect might be exaggerated. Larger sample RCTs are needed in the future for accurate results. 

Third, all the decoctions included in the research were based on Dachengqi Decoction, but the herbs and the dosages were different in each study. This might be the main reason leading to the significant heterogeneity. We can see in the research that all the studies except six pointed out that the syndrome of phlegm-heat obstructing lung is appropriate for MDD. Determination of treatment based on pathogenesis obtained through differentiation of symptoms is one of the most important characteristics in Chinese Medicine, so modification according to symptoms is needed during the treatment. Herbs of cold nature such as Gypsum Fibrosum or Radix Scutellariae are used to clear heat while diminishing sputum herbs such as Fructrs Trichosanthis, Semen Lepidii, and Herba Houttuyniae are commonly used in patients with abundance phlegm on the basic of Dachengqi Decoction. 

Despite of the methodological weakness and potential risk of bias, the data from the 16 included studies illustrated that MDD combined with conventional treatment may have better effectiveness than conventional treatment alone, especially in improving FEV_1_%pred, enhancing significant effectiveness, reducing PCO_2_, and shortening duration of mechanical ventilation. The study suggests that MDD could improve airway obstruction and relieve respiratory failure so as to improve prognosis. 

## 5. Conclusion

Modified Dachengqi Decoction appears to be effective for treating AECOPD. However, more well-designed and large sample RCTs are needed in the future due to the insufficient methodological problems of existing studies.

## Figures and Tables

**Table 1:** Characteristics of included trials.

Study ID	Sample	CM syndrome	Intervention	Controlled	Couse	Adverse event	Outcome measures
Fang and Shi, 2006 [9]	T: 20/18 C: 20/17	NS	MDD	Aminophylline 0.25 g qd, Methylprednisolone 80 mg q12h, Cefoperazone and Sulbactam 2 g q12h, Mucosolvan 75 mg q12h	5D	NS	BGA, DMV
Fu, 2010 [10]	T: 30/30 C: 30/30	SPHOL	MDD	Conventional treatment	9D	NS	PF
Guo and Liang, 2011 [11]	T: 60/60 C: 60/60	SPHOL	MDD	Mucosolvan 60 mg and conventional treatment	14D	NS	ER
Guo and Zhang, 2008 [12]	T: 30/30 C: 30/30	NS	MDD	Conventional treatment	7D	NS	PF, BGA, ER
Li, 2009 [13]	T: 29/29 C: 27/27	SPHOL	Tongsai granule 6 g tid	Aminophylline 0.5 g, antibacterial	15D	NS	Inflammation factor
Li et al., 2003 [14]	T: 60/60 C: 60/60	SPHOL	MDD	Conventional treatment	12D	NS	ER
Li, 2006 [15]	T: 30/30 C: 30/30	SPHOL	MDD	Cefaclor capsules 0.25 g tid, Azithromycin tablets 0.5 g, Mucosolvan 30 mg tid.	14D	YSE	PF, BGA, ER
Liang, 2011 [16]	T: 30/30 C: 30/30	NS	MDD	Conventional treatment	15D	NS	PF, ER
Liu et al., 2002 [17]	T: 25/25 C: 25/25	NS	MDD	Conventional treatment	12D	NS	Offline success rate
Lu, 2010 [18]	T: 21/21 C: 21/21	SPHOL	MDD	Conventional treatment	10D	NS	PF, BGA, ER
Mao, 2010 [19]	T: 52/52 C: 48/48	NS	Tongfupaiqi mixture	Conventional treatment	14D	YES	PF
Meng, 2012 [20]	T: 30/30 C: 30/30	SPHOL	MDD	Conventional treatment	7D	NS	PF, ER
Pang, 2009 [21]	T: 42/42 C: 40/40	SPHOL	MDD	Conventional treatment	7D	NS	BGA
Peng and Li, 2009 [22]	T: 30/30 C: 30/30	SPHOL	MDD	Conventional treatment	14D	NS	ER
Shi et al., 2010 [23]	T: 50/38 C: 30/20	NS	MDD	Conventional treatment	28D	NS	DMV
Zhang, 2011 [24]	T: 32/32 C: 30/30	SPHOL	MDD	Conventional treatment	7D	NS	PF, ER

T: treatment; C: control; NS: not specified; conventional treatment: antibiotics, antispasmodic, expectorant (the drug is unknown); SPHOL: syndrome of phlegm-heat obstructing lung; BGA: blood gas analysis. PF: pulmonary function; ER: effective rate; DMV: duration of mechanical ventilation.

**Table 2:** The compositions of MDD.

Study ID	Composition of formula
Fang and Shi, 2006 [9]	MDD (Radix et Rhizoma Rhei, Fructus Aurantii Immaturus, Cortex *Magnoliae officinalis*, Trichosanthis, Semen Armeniacae Amarum, Radix Glycytthizae, Semen Raphani.) 100 mL bid
Fu, 2010 [10]	MDD (Radix et Rhizoma Rhei, Fructus Aurantii Immaturus, Cortex *Magnoliae officinalis*) qd
Guo and Liang, 2011 [11]	MDD (Radix et Rhizoma Rhei, Fructus Aurantii Immaturus, Cortex *Magnoliae officinalis*, Rhizoma Pinelliae, Semen Raphani, Radix Astragali) 200 mL bid
Guo and Zhang, 2008 [12]	MDD (Gypsum Fibrosum, Radix Scutellariae, Semen Armeniacae Amarum, Fructrs Trichosanthis), 200 mL bid
Li, 2009 [13]	Tongsai granule (Radix et Rhizoma Rhei, Herba Ephedrae, Semen Lepidii, Bulbus Fritillariae Cirrhosae) 6 g tid
Li et al., 2003 [14]	MDD (Radix et Rhizoma Rhei, Fructus Aurantii Immaturus, Natrii Sulfas, Fructrs Trichosanthis, Semen Raphani, Semen Lepidii) 200 mL bid
Li, 2006 [15]	MDD (Radix et Rhizoma Rhei 10 g, Gypsum Fibrosum 30 g, Fructrs Trichosanthis 15 g, Semen Armeniacae Amarum 10 g, Radix Scutellariae 15 g, Semen Lepidii 15 g, Herba Houttuyniae 15 g, Bulbus Fritillariae Thunbergii 15 g, Radix Glycytthizae 15 g) 100 mL tid
Liang, 2011 [16]	MDD (Radix et Rhizoma Rhei 7 g, Fructus Aurantii Immaturus 9 g, Cortex *Magnoliae officinalis* 9 g, Radix Scutellariae 12 g, Rhizoma Pinelliae 14 g, Pericarpium Citri Reticulatae 11 g, Radix Glycytthizae 7 g) 200 mL bid
Liu et al., 2002 [17]	MDD (Radix et Rhizoma Rhei, Fructus Aurantii Immaturus, Cortex *Magnoliae officinalis*, Fructrs Trichosanthis, Semen Armeniacae Amarum, Poria, Radix Glycytthizae) 100 mL bid
Lu, 2010 [18]	MDD (Radix et Rhizoma Rhei 3 g, Fructrs Trichosanthis 15 g, Semen Armeniacae Amarum 10 g, Semen Cannabis 10 g, Radix Scutellariae 15 g, Herba Houttuyniae 15 g, Radix Glycytthizae 9 g) 200 mL bid
Mao, 2010 [19]	TongFuPaiQi mixture (Radix et Rhizoma Rhei, Semen Raphani, Semen Persicae, Radix Paeoniae Rubra) 25 mL tid
Meng, 2012 [20]	MDD (Radix et Rhizoma Rhei, Fructrs Trichosanthis, Semen Armeniacae Amarum Gypsum Fibrosum), 200 mL bid
Pang, 2009 [21]	MDD (Radix et Rhizoma Rhei, Cortex *Magnoliae officinalis*, Fructrs Trichosanthis, Semen Armeniacae Amarum, Rhizoma Pinelliae, Gypsum Fibrosum, Radix Astragali, Radix Glycytthizae) 200 mL bid
Peng and Li, 2009 [22]	MDD (Radix et Rhizoma Rhei 6 g, Natrii Sulfas 12 g, Semen Lepidii 15 g, Radix Scutellariae 15 g) qd
Shi et al., 2010 [23]	MDD (Radix et Rhizoma Rhei, Fructus Aurantii Immaturus, Fructrs Trichosanthis, Semen Armeniacae Amarum, Semen Raphani, Radix Glycytthizae) 100 mL bid
Zhang, 2011 [24]	MDD (Radix et Rhizoma Rhei, Cortex *Magnoliae officinalis*, Fructrs Trichosanthis, Semen Armeniacae Amarum, Rhizoma Pinelliae, Gypsum Fibrosum, Radix Scutellariae) 200 mL bid

**Table 3:** Outcome measures for PO_2_ (mmHg).

Study	Treatment (m ± s)	Control (m ± s)	Mean difference (95% CI)	*P* value
Fang and Shi, 2006 [9]	90 ± 21	68 ± 9	22.00 (11.40, 32.60)	*P* = 0.0004
Guo and Liang, 2011 [11]	82.3 ± 7.32	75.02 ± 8.34	7.28 (3.31, 11.25)	*P* = 0.0007
Li, 2009 [13]	80.25 ± 5.18	79.25 ± 7.36	1.00 (−2.22, 4.22)	*P* = 0.5452
Lu, 2010 [18]	92.95 ± 4.67	91.29 ± 5.02	1.66 (−1.70, 5.02)	*P* = 0.34
Meng, 2012 [20]	81.2 ± 4.9	75.2 ± 5.1	6.00 (3.47, 8.53)	*P* < 0.0001
Pang, 2009 [21]	69.87 ± 3.96	62.43 ± 4.14	7.44 (5.68, 9.20)	*P* < 0.0001
Meta			5.76 (4.60, 6.93)	*P* < 0.00001

**Table 4:** Outcome measures for PCO_2_ (mmHg).

Study	Treatment (m ± s)	Control (m ± s)	Mean difference (95% CI)	*P* value
Fang and Shi, 2006 [9]	67 ± 11	74 ± 13	−7.00 (−14.78, 0.78)	*P* = 0.0852
Guo and Liang, 2011 [11]	41.3 ± 5.13	48.26 ± 5.34	−6.96 (−9.61, − 4.31)	*P* < 0.0001
Lu, 2010 [18]	41.76 ± 2.1	42.76 ± 2.39	−1.00 (−2.56, 0.56)	*P* = 0.2184
Meng, 2012 [20]	65.7 ± 6.3	81.7 ± 8.1	−16.00 (−19.67, − 12.33)	*P* < 0.0001
Pang, 2009 [21]	57.54 ± 6.9	62.36 ± 5.76	−4.82 (−7.57, − 2.07)	*P* = 0.001
Meta			−4.30 (−5.44, − 3.17)	*P* < 0.00001
